# Supplementary material for: Population Genetic Study on the European Flounder (Platichthys flesus) from the Southern Baltic Sea Using SNPs and Microsatellite Markers
Source: Animals (Basel). 2023 Apr 24;13(9):1448. doi: 10.3390/ani13091448 (PMC10177365; doi:10.3390/ani13091448)
Supplement: Supplementary file 1 [file animals-13-01448-s001.zip › animals-2283839-supplementary.pdf]

**Supplementary Table S1.** Summary of utilized biological and morphological data in the present study. TL: total body length. SW: standard weight. LW: liver weight. *K*: Fulton's condition factor. *HSI*: hepatosomatic index. F: female. M: male.

| Sampling site | Biological and morphological trait | | | | | |
|---|---|---|---|---|---|---|
| | **Sex** | **SL (cm)** | **SW [g]** | **LW [g]** | ***K*** | ***HSI*** |
| Władysławowo | | | | | | |
| 1 | M | 16.6 | 48.0 | 0.74 | 1.05 | 1.54 |
| 2 | M | 18.9 | 76.0 | 0.94 | 1.13 | 1.24 |
| 3 | M | 19.5 | 77.6 | 0.98 | 1.05 | 1.26 |
| 4 | M | 22.0 | 155.0 | 3.70 | 1.46 | 2.39 |
| 5 | F | 26.0 | 216.0 | 5.64 | 1.23 | 2.61 |
| 6 | F | 26.0 | 256.0 | 7.46 | 1.46 | 2.91 |
| 7 | M | 17.0 | 51.9 | 0.36 | 1.06 | 0.69 |
| 8 | M | 18.5 | 71.2 | 1.38 | 1.12 | 1.94 |
| 9 | M | 18.7 | 71.5 | 1.46 | 1.09 | 2.04 |
| 10 | M | 19.2 | 73.6 | 1.61 | 1.04 | 2.19 |
| 11 | M | 20.5 | 88.7 | 1.29 | 1.03 | 1.45 |
| 12 | F | 26.0 | 226.0 | 4.57 | 1.29 | 2.02 |
| 13 | F | 28.0 | 276.0 | 6.49 | 1.26 | 2.35 |
| 14 | F | 27.0 | 305.0 | 5.55 | 1.55 | 1.82 |
| 15 | F | 28.0 | 328.0 | 8.32 | 1.49 | 2.54 |
| 16 | F | 29.0 | 335.0 | 8.94 | 1.37 | 2.67 |
| 17 | M | 19.4 | 72.2 | 1.20 | 0.99 | 1.66 |
| 18 | M | 20.4 | 78.2 | 0.80 | 0.92 | 1.02 |
| 19 | M | 19.0 | 81.0 | 0.91 | 1.18 | 1.12 |
| 20 | M | 20.1 | 90.0 | 2.64 | 1.11 | 2.93 |
| 21 | M | 20.2 | 90.7 | 1.61 | 1.10 | 1.78 |
| 22 | M | 21.1 | 90.8 | 2.39 | 0.97 | 2.63 |
| 23 | M | 20.3 | 91.4 | 2.30 | 1.09 | 2.52 |
| 24 | M | 20.2 | 93.5 | 1.54 | 1.13 | 1.65 |
| 25 | M | 21.0 | 95.2 | 2.60 | 1.03 | 2.73 |
| 26 | M | 21.0 | 97.0 | 1.58 | 1.05 | 1.63 |
| 27 | F | 26.0 | 241.0 | 5.26 | 1.37 | 2.18 |
| 28 | F | 26.0 | 243.0 | 4.65 | 1.38 | 1.91 |
| 29 | F | 28.0 | 267.0 | 5.63 | 1.22 | 2.11 |
| 30 | F | 27.0 | 274.0 | 6.68 | 1.39 | 2.44 |
| 31 | F | 28.0 | 290.0 | 7.17 | 1.32 | 2.47 |
| 32 | F | 28.0 | 297.0 | 8.43 | 1.35 | 2.84 |
| 33 | F | 29.1 | 297.8 | 8.16 | 1.21 | 2.74 |
| 34 | F | 28.0 | 300.0 | 10.24 | 1.37 | 3.41 |
| 35 | F | 30.0 | 302.0 | 7.36 | 1.12 | 2.44 |
| 36 | F | 29.0 | 327.0 | 9.56 | 1.34 | 2.92 |
| 37 | M | 24.7 | 135.3 | 2.00 | 0.90 | 1.48 |
| 38 | F | 31.0 | 443.0 | 9.86 | 1.49 | 2.23 |
| 39 | F | 27.7 | 206.9 | 2.20 | 0.97 | 1.06 |

| | | | | | | |
|---|---|---|---|---|---|---|
| 40 | F | 27.0 | 247.0 | 4.60 | 1.25 | 1.86 |
| 41 | F | 32.0 | 366.0 | 11.27 | 1.12 | 3.08 |
| 42 | M | 23.0 | 107.0 | 1.62 | 0.88 | 1.51 |
| 43 | F | 30.0 | 223.2 | 2.40 | 0.83 | 1.08 |
| 44 | F | 29.0 | 265.0 | 10.30 | 1.09 | 3.89 |
| 45 | M | 29.0 | 241.0 | 4.01 | 0.99 | 1.66 |
| 46 | F | 30.0 | 287.0 | 7.81 | 1.06 | 2.72 |
| 47 | F | 33.0 | 327.0 | 4.30 | 0.91 | 1.31 |
| 48 | F | 35.0 | 458.0 | 11.32 | 1.07 | 2.47 |
| 49 | F | 34.0 | 470.6 | 14.44 | 1.20 | 3.07 |
| 50 | M | 34.0 | 380.0 | 5.51 | 0.97 | 1.45 |
| Mechelinki | | | | | | |
| 1 | M | 15.8 | 34.6 | 0.40 | 0.88 | 1.16 |
| 2 | M | 18.0 | 63.0 | 0.68 | 1.08 | 1.08 |
| 3 | F | 20.7 | 88.0 | 0.97 | 0.99 | 1.10 |
| 4 | F | 20.7 | 88.0 | 0.97 | 0.99 | 1.10 |
| 5 | M | 22.0 | 116.0 | 3.16 | 1.09 | 2.72 |
| 6 | M | 24.0 | 168.0 | 2.43 | 1.22 | 1.45 |
| 7 | M | 18.0 | 58.0 | 0.80 | 0.99 | 1.38 |
| 8 | M | 18.7 | 71.0 | 1.01 | 1.09 | 1.42 |
| 9 | M | 18.7 | 81.0 | 1.07 | 1.24 | 1.32 |
| 10 | M | 19.0 | 69.0 | 0.95 | 1.01 | 1.38 |
| 11 | M | 19.5 | 82.0 | 1.73 | 1.11 | 2.11 |
| 12 | F | 23.0 | 161.0 | 4.69 | 1.32 | 2.91 |
| 13 | F | 24.0 | 197.0 | 3.75 | 1.43 | 1.90 |
| 14 | F | 25.0 | 210.0 | 3.47 | 1.34 | 1.65 |
| 15 | F | 26.0 | 200.0 | 5.30 | 1.14 | 2.65 |
| 16 | M | 26.0 | 222.0 | 2.76 | 1.26 | 1.24 |
| 17 | F | 26.9 | 219.0 | 3.85 | 1.13 | 1.76 |
| 18 | F | 27.0 | 276.0 | 5.68 | 1.40 | 2.06 |
| 19 | F | 28.0 | 286.0 | 6.78 | 1.30 | 2.37 |
| 20 | F | 29.0 | 298.0 | 6.61 | 1.22 | 2.22 |
| 21 | F | 30.0 | 327.0 | 8.19 | 1.21 | 2.50 |
| 22 | F | 30.5 | 244.0 | 3.30 | 0.86 | 1.35 |
| 23 | M | 16.0 | 39.2 | 0.60 | 0.96 | 1.53 |
| 24 | M | 16.1 | 47.0 | 0.94 | 1.13 | 2.00 |
| 25 | M | 16.8 | 51.0 | 0.74 | 1.08 | 1.45 |
| 26 | M | 17.2 | 55.9 | 0.30 | 1.10 | 0.54 |
| 27 | M | 17.8 | 45.7 | 0.20 | 0.81 | 0.44 |
| 28 | M | 18.3 | 72.0 | 1.13 | 1.17 | 1.57 |
| 29 | M | 19.0 | 66.1 | 1.10 | 0.96 | 1.66 |
| 30 | M | 19.3 | 75.0 | 1.38 | 1.04 | 1.84 |
| 31 | M | 19.6 | 79.6 | 1.00 | 1.06 | 1.26 |
| 32 | M | 19.9 | 79.0 | 1.42 | 1.00 | 1.80 |
| 33 | F | 25.6 | 220.0 | 3.73 | 1.31 | 1.70 |
| 34 | F | 27.0 | 218.0 | 3.38 | 1.11 | 1.55 |

| | | | | | | |
|---|---|---|---|---|---|---|
| 35 | F | 27.0 | 234.0 | 4.18 | 1.19 | 1.79 |
| 36 | F | 27.0 | 238.0 | 4.21 | 1.21 | 1.77 |
| 37 | F | 27.0 | 239.0 | 5.05 | 1.21 | 2.11 |
| 38 | F | 27.0 | 250.0 | 4.27 | 1.27 | 1.71 |
| 39 | F | 27.1 | 236.0 | 4.15 | 1.19 | 1.76 |
| 40 | F | 27.6 | 258.0 | 7.78 | 1.23 | 3.02 |
| 41 | F | 29.0 | 290.0 | 3.83 | 1.19 | 1.32 |
| 42 | F | 30.0 | 345.0 | 7.23 | 1.28 | 2.10 |
| 43 | M | 22.7 | 155.0 | 3.59 | 1.33 | 2.32 |
| 44 | M | 24.0 | 125.0 | 4.51 | 0.90 | 3.61 |
| 45 | F | 31.0 | 325.0 | 8.92 | 1.09 | 2.74 |
| 46 | F | 29.0 | 223.0 | 3.80 | 0.91 | 1.70 |
| 47 | M | 26.1 | 150.0 | 2.73 | 0.84 | 1.82 |
| 48 | F | 32.0 | 381.0 | 9.43 | 1.16 | 2.48 |
| 49 | M | 31.0 | 343.0 | 6.88 | 1.15 | 2.01 |
| 50 | F | 36.0 | 565.0 | 16.19 | 1.21 | 2.87 |
| Słupsk Bank | | | | | | |
| 1 | M | 18.5 | 74.0 | 1.0 | 1.17 | 1.35 |
| 2 | F | 21.0 | 95.0 | 2.0 | 1.03 | 2.11 |
| 3 | M | 22.0 | 100.0 | 1.0 | 0.94 | 1.00 |
| 4 | M | 23.0 | 152.0 | 1.0 | 1.25 | 0.66 |
| 5 | M | 23.0 | 130.0 | 1.0 | 1.07 | 0.77 |
| 6 | F | 25.0 | 165.0 | 2.0 | 1.06 | 1.21 |
| 7 | M | 28.0 | 235.0 | 1.0 | 1.07 | 0.43 |
| 8 | M | 22.0 | 132.0 | 1.0 | 1.24 | 0.76 |
| 9 | M | 22.0 | 130.0 | 1.0 | 1.22 | 0.77 |
| 10 | M | 23.0 | 140.0 | 1.0 | 1.15 | 0.71 |
| 11 | M | 23.0 | 114.0 | 1.0 | 0.94 | 0.88 |
| 12 | M | 23.0 | 158.0 | 1.0 | 1.30 | 0.63 |
| 13 | F | 24.0 | 138.0 | 2.0 | 1.00 | 1.45 |
| 14 | M | 24.0 | 170.0 | 1.0 | 1.23 | 0.59 |
| 15 | M | 24.0 | 150.0 | 1.0 | 1.09 | 0.67 |
| 16 | M | 24.0 | 148.0 | 1.0 | 1.07 | 0.68 |
| 17 | F | 24.0 | 182.0 | 2.0 | 1.32 | 1.10 |
| 18 | F | 24.0 | 160.0 | 2.0 | 1.16 | 1.25 |
| 19 | M | 25.0 | 190.0 | 1.0 | 1.22 | 0.53 |
| 20 | M | 25.0 | 160.0 | 1.0 | 1.02 | 0.63 |
| 21 | M | 25.0 | 155.0 | 1.0 | 0.99 | 0.65 |
| 22 | M | 25.0 | 180.0 | 1.0 | 1.15 | 0.56 |
| 23 | M | 25.0 | 176.0 | 1.0 | 1.13 | 0.57 |
| 24 | F | 25.0 | 210.0 | 2.0 | 1.34 | 0.95 |
| 25 | M | 25.0 | 182.0 | 1.0 | 1.16 | 0.55 |
| 26 | M | 26.0 | 170.0 | 1.0 | 0.97 | 0.59 |
| 27 | M | 26.0 | 204.0 | 1.0 | 1.16 | 0.49 |
| 28 | M | 26.0 | 198.0 | 1.0 | 1.13 | 0.51 |
| 29 | M | 26.0 | 202.0 | 1.0 | 1.15 | 0.50 |

| | | | | | | |
|---|---|---|---|---|---|---|
| 30 | M | 27.0 | 206.0 | 1.0 | 1.05 | 0.49 |
| 31 | M | 27.0 | 220.0 | 1.0 | 1.12 | 0.45 |
| 32 | M | 27.0 | 180.0 | 1.0 | 0.91 | 0.56 |
| 33 | M | 28.0 | 208.0 | 1.0 | 0.95 | 0.48 |
| 34 | M | 29.0 | 215.0 | 1.0 | 0.88 | 0.47 |
| 35 | M | 30.0 | 280.0 | 1.0 | 1.04 | 0.36 |
| 36 | F | 27.0 | 225.0 | 2.0 | 1.14 | 0.89 |
| 37 | M | 27.0 | 218.0 | 1.0 | 1.11 | 0.46 |
| 38 | M | 28.0 | 285.0 | 1.0 | 1.30 | 0.35 |
| 39 | F | 28.0 | 214.0 | 2.0 | 0.97 | 0.93 |
| 40 | F | 29.0 | 280.0 | 2.0 | 1.15 | 0.71 |
| 41 | M | 29.0 | 290.0 | 1.0 | 1.19 | 0.34 |
| 42 | M | 29.0 | 266.0 | 1.0 | 1.09 | 0.38 |
| 43 | F | 30.0 | 302.0 | 2.0 | 1.12 | 0.66 |
| 44 | F | 30.0 | 270.0 | 2.0 | 1.00 | 0.74 |
| 45 | F | 31.0 | 266.0 | 2.0 | 0.89 | 0.75 |
| 46 | F | 32.0 | 336.0 | 2.0 | 1.03 | 0.60 |
| 47 | F | 31.0 | 354.0 | 2.0 | 1.19 | 0.56 |
| 48 | F | 30.0 | 358.0 | 2.0 | 1.33 | 0.56 |
| 49 | F | 29.0 | 395.0 | 2.0 | 1.62 | 0.51 |
| 50 | M | 30.0 | 300.0 | 1.0 | 1.11 | 0.33 |
| Vistula Lagoon | | | | | | |
| 1 | M | 18 | 89 | - | 1.53 | - |
| 2 | F | 24 | 162 | - | 1.17 | - |
| 3 | M | 17 | 64 | - | 1.30 | - |
| 4 | M | 24 | 178 | - | 1.29 | - |
| 5 | M | 15 | 43 | - | 1.27 | - |
| 6 | M | 23 | 162 | - | 1.33 | - |
| 7 | F | 17 | 64 | - | 1.30 | - |
| 8 | M | 18 | 74 | - | 1.27 | - |
| 9 | F | 16 | 52 | - | 1.27 | - |
| 10 | F | 16 | 57 | - | 1.39 | - |
| 11 | M | 23 | 159 | - | 1.31 | - |
| 12 | M | 21 | 128 | - | 1.38 | - |
| 13 | F | 16 | 49 | - | 1.20 | - |
| 14 | F | 13 | 31 | - | 1.41 | - |
| 15 | M | 14 | 39 | - | 1.42 | - |
| 16 | M | 16 | 49 | - | 1.20 | - |
| 17 | M | 15 | 42 | - | 1.24 | - |
| 18 | M | 15 | 43 | - | 1.27 | - |
| 19 | F | 16 | 54 | - | 1.32 | - |
| 20 | F | 14 | 37 | - | 1.35 | - |
| 21 | F | 15 | 42 | - | 1.24 | - |
| 22 | F | 15 | 48 | - | 1.42 | - |
| 23 | M | 14 | 38 | - | 1.38 | - |
| 24 | M | 14 | 39 | - | 1.42 | - |

| | | | | | | |
|---|---|---|---|---|---|---|
| 25 | F | 13 | 41 | - | 1.87 | - |
| 26 | M | 14 | 36 | - | 1.31 | - |
| 27 | M | 14 | 39 | - | 1.42 | - |
| 28 | M | 13 | 28 | - | 1.27 | - |
| 29 | M | 17 | 64 | - | 1.30 | - |
| 30 | F | 13 | 32 | - | 1.46 | - |
| 31 | F | 19 | 76 | - | 1.11 | - |
| 32 | F | 13 | 30 | - | 1.37 | - |
| 33 | M | 13 | 28 | - | 1.27 | - |
| 34 | M | 12 | 29 | - | 1.68 | - |
| 35 | F | 17 | 59 | - | 1.20 | - |
| 36 | M | 17 | 68 | - | 1.38 | - |
| 37 | F | 18 | 78 | - | 1.34 | - |
| 38 | M | 12 | 23 | - | 1.33 | - |
| 39 | F | 17 | 56 | - | 1.14 | - |
| 40 | M | 15 | 52 | - | 1.54 | - |

**Supplementary Table S2.** Characterization of ten microsatellite loci used in the present study: locus designation (Locus), NCBI accession number (Acc. num.), primer concentrations in the reaction mix (Conc.), fluorescence dye (Dye), allele size range (Size), number of observed alleles (*Ao*). F – forward 5′-tailed primer, M13 – 5′-fluorescently labelled universal primer, R – reverse primer.

| Locus | Acc. num. | Conc. (µM) | Dye | Size | *Ao* | References |
|---|---|---|---|---|---|---|
| **<u>Multiplex I</u>** | | | | | | |
| *StPf1022* | AJ538320 | F: 0.025/M13: 0.104<br>R: 0.125 | ROX | 182–232 | 18 | [15] |
| *PL142* | AF406750 | F: 0.008/M13: 0.033<br>R: 0.040 | HEX | 161–221 | 24 | [15] |
| *FLAG5-83* | FJ360511 | F: 0.012/M13: 0.049<br>R: 0.060 | FAM | 223–377 | 47 | [17] |
| *FLAG4-71* | FJ360510 | F: 0.016/M13: 0.066<br>R: 0.08 | HEX | 187–253 | 28 | [17] |
| **<u>Multiplex II</u>** | | | | | | |
| *FLAG2-76* | FJ360505 | F: 0.034/M13: 0.141<br>R: 0.170 | CY3 | 175–249 | 28 | [17] |
| *FLAG4-65* | FJ360509 | F: 0.026/M13: 0.108<br>R: 0.130 | HEX | 223–357 | 36 | [17] |
| *FLAG8-37* | FJ360517 | F: 0.025/M13: 0.104<br>R:0.125 | ROX | 172–284 | 27 | [17] |
| *Nplaf-33* | KT354915 | F: 0.013/M13: 0.054<br>R:0.065 | FAM | 268–520 | 19 | [18] |
| **<u>Multiplex III</u>** | | | | | | |
| *StPf1006* | AJ315972 | F: 0.029/M13: 0.120 | FAM | 254–398 | 36 | [16] |

| | | | | | | |
|---|---|---|---|---|---|---|
| | | R: 0.145 | | | | |
| *StPf1001* | AJ315970 | F: 0.008/M13: 0.033<br>R: 0.040 | HEX | 251–365 | 29 | [15] |
| *FLAC4-67* | FJ360499 | F: 0.033/M13: 0.137<br>R: 0.165 | CY3 | 177–261 | 34 | [17] |
| *FLAC4–69* | FJ360500 | F: 0.01/M13: 0.045<br>R: 0.050 | ROX | 204–310 | 26 | [17] |
| **<u>Multiplex IV</u>** | | | | | | |
| *PL167* | AF406751 | F: 0.034/M13: 0.141<br>R: 0.170 | CY3 | 186–250 | 18 | [15] |
| *StPf1004* | AJ315973 | F: 0.008/M13: 0.033<br>R: 0.040 | HEX | 150–218 | 19 | [15] |
| *StPf1003* | AJ315971 | F: 0.013/M13: 0.054<br>R:0.065 | FAM | 187–207 | 12 | [16] |
| *StPf1016* | AJ538314 | F: 0.025/M13: 0.104<br>R:0.125 | ROX | 146–192 | 32 | [16] |
| **<u>Multiplex V</u>** | | | | | | |
| *StPf1002* | AJ315975 | F: 0.025/M13: 0.104<br>R:0.125 | HEX | 187–203 | 7 | [15] |
| *StPf1015* | AJ538313 | F: 0.013/M13: 0.054<br>R:0.065 | FAM | 134–158 | 6 | [15] |
| *StPf1005* | AJ315974 | F: 0.034/M13: 0.141<br>R: 0.170 | CY3 | 115–135 | 9 | [15] |

**Supplementary Table S3** The scheme of fish division on series of separate groups according to their sex, age and calculated values of Fulton's condition factor (*K*) and hepatosomatic index (*HSI*) for the analysis of correspondence between biological and morphological traits as well as genetic characteristics.

| Number of groups | Discriminating trait and its characteristics | | |
|---|---|---|---|
| | **Sex** | ***K*** | ***HSI*** |
| 1 | Female (*n* = 86) | <1.00 (*n* = 30) | <0.66 (*n* = 30) |
| 2 | Male (*n* = 104) | 1.00-1.09 (*n* = 32) | 0.66-1,25 (*n* = 30) |
| 3 | - | 1.10-1.16 (*n* = 30) | 1.26-1.71 (*n* = 30) |
| 4 | - | 1.17-1.26 (*n* = 38) | 1.76-2.37 (*n* = 30) |
| 5 | - | >1.26 (*n* = 60) | >2.37 (*n* = 30) |

**Supplementary Table S4.** Genetic diversity parameters of the studied European flounder (*Platichthys flesus*) individuals under the current study. *Ao*: observed number of alleles, *Ae*: number of effective alleles, *Ho*: observed and *He*: expected heterozygosity, *I*: Shannon's index, *PIC*: polymorphism information content, *Fis*: fixation index. Values of H-WE test and values of *Fis* indicator significant at: a $p<0.05$, b $p<0.01$, c $p<0.001$. Significant values of H-WE test and values of *Fis* indicator after Bonferroni correction were bolded.

| **Sampling site/ locus** | ***Ao*** | ***Ae*** | ***Ho*** | ***He*** | ***p*** | ***I*** | ***PIC*** | ***Fis*** |
|---|---|---|---|---|---|---|---|---|
| Władysławowo | | | | | | | | |
| *StPf1022* | 15 | 6 | 0.854 | 0.871 | 0.281 | 2.086 | 0.839 | 0.017 |
| *PL142* | 20 | 12 | 0.933 | 0.919 | 0.763 | 2.566 | 0.897 | -0.014 |
| *FLAG5-83* | 33 | 21 | 0.960 | 0.962 | 0.342 | 3.226 | 0.950 | 0.002 |
| *FLAG4-71* | 24 | 14 | 0.920 | 0.937 | 0.883 | 2.858 | 0.924 | 0.019 |
| *FLAG2-76* | 20 | 10 | 0.820 | 0.913 | 0.014[b] | 2.571 | 0.896 | 0.103[a] |
| *FLAG4-65* | 28 | 17 | 0.920 | 0.950 | 0.364 | 3.025 | 0.937 | 0.032 |
| *FLAG8-37* | 19 | 9 | 0.720 | 0.894 | 0.001[b] | 2.486 | 0.875 | 0.195[b] |
| *Nplaf_33* | 14 | 6 | 0.880 | 0.828 | 0.276 | 2.077 | 0.803 | -0.064 |
| *StPf1006* | 30 | 20 | 0.880 | 0.961 | 0.141 | 3.181 | 0.949 | 0.085[b] |
| *StPf1001* | 20 | 4 | 0.780 | 0.767 | 0.506 | 2.038 | 0.743 | -0.017 |
| *FLAC4-67* | 30 | 14 | 0.820 | 0.940 | 0.002[b] | 2.983 | 0.926 | 0.128[b] |
| *FLAC4–69* | 20 | 8 | 0.840 | 0.886 | 0.497 | 2.522 | 0.869 | 0.052 |
| *PL167* | 15 | 8 | 0.821 | 0.841 | 0.0152 | 2.094 | 0.801 | 0.020 |
| *StPf1004* | 13 | 5 | 0.689 | 0.714 | 0.478 | 2.238 | 0.723 | 0.025 |
| *StPf1003* | 10 | 4 | 0.665 | 0.600 | 0.935 | 2.131 | 0.605 | -0.065 |
| *StPf1016* | 28 | 15 | 0.801 | 0.853 | 0.555 | 2.987 | 0.837 | 0.052 |
| *StPf1002* | 6 | 4 | 0.599 | 0.696 | 0.027[b] | 2.075 | 0.743 | 0.097[a] |
| *StPf1015* | 6 | 3 | 0.504 | 0.522 | 0.641 | 2.236 | 0.698 | 0.017 |
| *StPf1005* | 7 | 3 | 0.586 | 0.612 | 0.5377 | 2.163 | 0.726 | 0.025 |

| | | | | | | | | |
|---|---|---|---|---|---|---|---|---|
| Average | 18.8 | 9.6 | 0.815 | 0.847 | 0.121 | 2.697 | 0.887 | 0.031 |
| | | | | Mechelinki | | | | |
| *StPf1022* | 13 | 8 | 0.861 | 0.882 | 0.357 | 2.531 | 0.914 | 0.021 |
| *PL142* | 19 | 10 | 0.889 | 0.914 | 0.745 | 2.931 | 0.918 | 0.025 |
| *FLAG5-83* | 34 | 19 | 0.860 | 0.957 | 0.070 | 3.224 | 0.952 | 0.102 [b] |
| *FLAG4-71* | 24 | 14 | 0.840 | 0.939 | 0.059 | 2.877 | 0.928 | 0.106 [a] |
| *FLAG2-76* | 19 | 11 | 0.880 | 0.917 | 0.062 | 2.607 | 0.921 | 0.040 |
| *FLAG4-65* | 24 | 18 | 0.940 | 0.953 | 0.318 | 3.000 | 0.948 | 0.013 |
| *FLAG8-37* | 17 | 9 | 0.740 | 0.894 | 0.001 [c] | 2.410 | 0.897 | 0.174 [b] |
| *Nplaf_33* | 15 | 7 | 0.760 | 0.861 | 0.069 | 2.200 | 0.847 | 0.118 [a] |
| *StPf1006* | 29 | 18 | 0.880 | 0.953 | 0.036 [a] | 3.094 | 0.947 | 0.077 |
| *StPf1001* | 21 | 5 | 0.760 | 0.801 | 0.166 | 2.166 | 0.769 | 0.051 |
| *FLAC4-67* | 28 | 17 | 0.840 | 0.949 | 0.004 [b] | 3.039 | 0.936 | 0.116 [b] |
| *FLAC4–69* | 17 | 8 | 0.900 | 0.889 | 0.958 | 2.477 | 0.905 | -0.011 |
| *PL167* | 13 | 6 | 0.824 | 0.868 | 0.046 [a] | 2.897 | 0.846 | 0.045 |
| *StPf1004* | 14 | 7 | 0.607 | 0.716 | 0.162 | 2.561 | 0.785 | 0.109 [b] |
| *StPf1003* | 9 | 4 | 0.745 | 0.685 | 0.945 | 2.791 | 0.797 | -0.030 |
| *StPf1016* | 26 | 12 | 0.785 | 0.875 | 0.165 | 2.862 | 0.764 | 0.090 |
| *StPf1002* | 5 | 3 | 0.594 | 0.687 | 0.027 [b] | 2.156 | 0.652 | 0.093 [a] |
| *StPf1015* | 6 | 4 | 0.472 | 0.436 | 0.630 | 2.284 | 0.521 | -0.036 |
| *StPf1005* | 8 | 3 | 0.587 | 0.598 | 0.335 | 2.365 | 0.623 | 0.011 |
| Average | 17.9 | 9.6 | 0.767 | 0.841 | 0.045 [a] | 2.656 | 0.835 | 0.074 [a] |
| | | | | Słupsk Bank | | | | |
| *StPf1022* | 14 | 7 | 0.876 | 0.888 | 0.662 | 2.368 | 0.893 | 0.012 |

| | | | | | | | | |
|---|---|---|---|---|---|---|---|---|
| *PL142* | 22 | 13 | 0.904 | 0.908 | 0.906 | 2.923 | 0.834 | 0.004 |
| *FLAG5-83* | 35 | 18 | 0.860 | 0.954 | 0.007 [b] | 3.194 | 0.943 | 0.100 [b] |
| *FLAG4-71* | 24 | 14 | 0.940 | 0.936 | 0.873 | 2.875 | 0.923 | -0.004 |
| *FLAG2-76* | 25 | 16 | 0.920 | 0.947 | 0.133 | 2.948 | 0.934 | 0.029 |
| *FLAG4-65* | 26 | 19 | 0.980 | 0.955 | 0.67 | 3.042 | 0.944 | -0.026 |
| *FLAG8-37* | 20 | 10 | 0.500 | 0.913 | **0.000** [c] | 2.583 | 0.897 | **0.455** [c] |
| *Nplaf_33* | 14 | 7 | 0.840 | 0.871 | 0.268 | 2.262 | 0.848 | 0.045 |
| *StPf1006* | 29 | 18 | 0.920 | 0.955 | 0.314 | 3.101 | 0.943 | 0.037 |
| *StPf1001* | 16 | 4 | 0.720 | 0.783 | 0.374 | 1.994 | 0.757 | 0.082 |
| *FLAC4-67* | 28 | 14 | 0.820 | 0.940 | 0.057 | 2.94 | 0.927 | 0.129 [b] |
| *FLAC4–69* | 21 | 13 | 0.880 | 0.933 | 0.218 | 2.743 | 0.918 | 0.057 |
| *PL167* | 17 | 8 | 0.772 | 0.832 | 0.036 [a] | 2.312 | 0.782 | 0.060 |
| *StPf1004* | 18 | 9 | 0.529 | 0.664 | 0.005 [b] | 2.268 | 0.654 | 0.135 [b] |
| *StPf1003* | 11 | 5 | 0.675 | 0.645 | 0.690 | 2.113 | 0.723 | -0.030 |
| *StPf1016* | 27 | 18 | 0.775 | 0.890 | 0.085 | 3.087 | 0.723 | 0.115 [a] |
| *StPf1002* | 7 | 4 | 0.566 | 0.688 | 0.021 [a] | 2.023 | 0.623 | 0.122 [b] |
| *StPf1015* | 6 | 3 | 0.492 | 0.464 | 0.679 | 1.978 | 0.589 | -0.028 |
| *StPf1005* | 9 | 5 | 0.624 | 0.600 | 0.459 | 2.036 | 0.644 | -0.024 |
| Average | 19.4 | 10.8 | 0.751 | 0.844 | **0.005** [b] | 2.768 | 0.903 | 0.093 [b] |
| Vistula lagoon | | | | | | | | |
| *StPf1022* | 16 | 9 | 0.903 | 0.896 | 0.811 | 2.309 | 0.877 | -0.007 |
| *PL142* | 20 | 12 | 0.886 | 0.935 | 0.237 | 2.749 | 0.908 | 0.049 |
| *FLAG5-83* | 34 | 19 | 0.910 | 0.960 | 0.206 | 3.225 | 0.951 | 0.050 |
| *FLAG4-71* | 24 | 14 | 0.880 | 0.938 | 0.471 | 2.868 | 0.926 | 0.058 |

| | | | | | | | | |
|---|---|---|---|---|---|---|---|---|
| *FLAG2-76* | 21 | 12 | 0.850 | 0.915 | 0.038 | 2.589 | 0.909 | 0.045 |
| *FLAG4-65* | 26 | 18 | 0.930 | 0.952 | 0.341 | 3.013 | 0.943 | 0.022 |
| *FLAG8-37* | 19 | 9 | 0.730 | 0.894 | 0.001 [b] | 2.448 | 0.886 | 0.164 [b] |
| *Nplaf_33* | 14 | 7 | 0.820 | 0.845 | 0.173 | 2.139 | 0.825 | 0.025 |
| *StPf1006* | 29 | 19 | 0.880 | 0.957 | 0.089 | 3.138 | 0.948 | 0.077 |
| *StPf1001* | 19 | 4 | 0.770 | 0.784 | 0.336 | 2.102 | 0.756 | 0.014 |
| *FLAC4-67* | 29 | 15 | 0.830 | 0.945 | 0.003 [b] | 3.011 | 0.931 | 0.115 [b] |
| *FLAC4–69* | 19 | 10 | 0.870 | 0.888 | 0.728 | 2.500 | 0.887 | 0.018 |
| *PL167* | 16 | 7 | 0.769 | 0.829 | 0.181 | 2.496 | 0.824 | 0.060 |
| *StPf1004* | 17 | 9 | 0.560 | 0.691 | 0.010 [a] | 2.400 | 0.754 | 0.131 [b] |
| *StPf1003* | 8 | 3 | 0.695 | 0.770 | 0.225 | 2.461 | 0.701 | 0.075 |
| *StPf1016* | 25 | 14 | 0.630 | 0.785 | 0.035 [a] | 2.925 | 0.801 | 0.155 [b] |
| *StPf1002* | 6 | 3 | 0.730 | 0.690 | 0.790 | 2.116 | 0.698 | -0.040 |
| *StPf1015* | 6 | 3 | 0.486 | 0.480 | 0.525 | 2.260 | 0.610 | -0.006 |
| *StPf1005* | 8 | 4 | 0.687 | 0.653 | 0.567 | 2.264 | 0.675 | -0.034 |
| Average | 18.8 | 10.1 | 0.801 | 0.844 | 0.214 | 2.579 | 0.832 | 0.036 |

**Supplementary Table S5.** Genotyping results of ten randomly selected European flounders from each sampled area located across southern Baltic Sea by two diagnostic SNP markers according to data published by Momigliano et al., (2017, 2019).

| **Sampling site/ fish number** | **Marker SNP 886_19** | **Marker SNP 3599_4** | **Identified species** |
|---|---|---|---|
| | Władysławowo | | |
| 1 | GG | CC | *P. flesus* |
| 2 | GG | CC | *P. flesus* |

| | | | |
|---|---|---|---|
| 3 | GG | CC | *P. flesus* |
| 4 | AG | CC | *P. flesus* |
| 5 | GG | CC | *P. flesus* |
| 6 | GG | CC | *P. flesus* |
| 7 | AG | CC | *P. flesus* |
| 8 | GG | CC | *P. flesus* |
| 9 | GG | CC | *P. flesus* |
| 10 | GG | CC | *P. flesus* |
| Mechelinki | | | |
| 1 | GG | CC | *P. flesus* |
| 2 | GG | CC | *P. flesus* |
| 3 | GG | CC | *P. flesus* |
| 4 | AA | CT | *P. flesus* |
| 5 | GG | CC | *P. flesus* |
| 6 | GG | CC | *P. flesus* |
| 7 | GG | CC | *P. flesus* |
| 8 | AG | CC | *P. flesus* |
| 9 | GG | CC | *P. flesus* |
| 10 | GG | CC | *P. flesus* |
| Słupsk Bank | | | |
| 1 | GG | CC | *P. flesus* |
| 2 | GG | CC | *P. flesus* |
| 3 | AA | CC | *P. flesus* |
| 4 | GG | CC | *P. flesus* |

| | | | |
|---|---|---|---|
| 5 | GG | CC | *P. flesus* |
| 6 | AG | CC | *P. flesus* |
| 7 | GG | CC | *P. flesus* |
| 8 | GG | CT | *P. flesus* |
| 9 | GG | CC | *P. flesus* |
| 10 | AA | CC | *P. flesus* |
| | Vistula Lagoon | | |
| 1 | GG | CC | *P. flesus* |
| 2 | AA | CC | *P. flesus* |
| 3 | GG | CC | *P. flesus* |
| 4 | GG | CC | *P. flesus* |
| 5 | GG | CC | *P. flesus* |
| 6 | GG | CC | *P. flesus* |
| 7 | GG | CC | *P. flesus* |
| 8 | GG | CC | *P. flesus* |
| 9 | GA | CC | *P. flesus* |
| 10 | GG | CC | *P. flesus* |

**Supplementary Table S6.** The obtained results from Bottleneck software analysis. Comparison of expected heterozygosity (*He*) vs heterozygosity (*Heq*) expected under an Infinite Allele Model (*IAM*). Stepwise Mutation Model (*SMM*) and two-phase model of mutation (*TPM*) in examined European flounder (*Platichthys flesus*) from the Baltic Sea. *M*: Garza-Williamson Index, Significant values of Wilcoxon test for *He*>*Heq* are bolded (*p*<0.05).

| **Locus/ fish group** | ***He*** | ***IAM*** | | ***TPM*** | | ***SMM*** | | ***M*** |
|---|---|---|---|---|---|---|---|---|
| | | ***Heq*** | ***p*** | ***Heq*** | ***p*** | ***Heq*** | ***p*** | |
| | | | | Władysławowo | | | | |
| *StPf1022* | 0.871 | 0.854 | 0.082 | 0.867 | 0.441 | 0.872 | 0.272 | 0.603 |

| | | | | | | | | |
|---|---|---|---|---|---|---|---|---|
| *PL142* | 0.919 | 0.892 | 0.153 | 0.915 | 0.450 | 0.925 | 0.139 | 0.642 |
| *FLAG5-83* | 0.962 | 0.945 | 0.060 | 0.958 | 0.420 | 0.963 | 0.351 | 0.478 |
| *FLAG4-71* | 0.937 | 0.910 | 0.103 | 0.933 | 0.461 | 0.943 | 0.193 | 0.727 |
| *FLAG2-76* | 0.913 | 0.884 | 0.202 | 0.913 | 0.439 | 0.929 | 0.085 | 0.556 |
| *FLAG4-65* | 0.950 | 0.928 | 0.106 | 0.946 | 0.481 | 0.953 | 0.237 | 0.418 |
| *FLAG8-37* | 0.894 | 0.874 | 0.381 | 0.908 | 0.222 | 0.924 | 0.027 | 0.339 |
| *Nplaf_33* | 0.828 | 0.815 | 0.480 | 0.865 | 0.119 | 0.893 | 0.012 | 0.933 |
| *StPf1006* | 0.961 | 0.936 | 0.004 | 0.951 | 0.141 | 0.957 | 0.456 | 0.938 |
| *StPf1001* | 0.767 | 0.883 | 0.014 | 0.914 | 0.001 | 0.930 | 0.003 | 0.400 |
| *FLAC4-67* | 0.940 | 0.936 | 0.535 | 0.951 | 0.092 | 0.957 | 0.024 | 0.714 |
| *FLAC4–69* | 0.886 | 0.884 | 0.418 | 0.914 | 0.094 | 0.929 | 0.006 | 0.404 |
| *PL167* | 0.841 | 0.812 | 0.106 | 0.841 | 0.435 | 0.857 | 0.245 | 0.560 |
| *StPf1004* | 0.714 | 0.692 | 0.082 | 0.710 | 0.441 | 0.717 | 0.272 | 0.603 |
| *StPf1003* | 0.600 | 0.580 | 0.153 | 0.614 | 0.450 | 0.630 | 0.139 | 0.642 |
| *StPf1016* | 0.853 | 0.840 | 0.154 | 0.890 | 0.460 | 0.918 | 0.161 | 0.487 |
| *StPf1002* | 0.696 | 0.671 | 0.030 | 0.686 | 0.352 | 0.692 | 0.132 | 0.379 |
| *StPf1015* | 0.522 | 0.638 | 0.431 | 0.669 | 0.171 | 0.685 | 0.020 | 0.636 |
| *StPf1005* | 0.612 | 0.608 | 0.242 | 0.623 | 0.130 | 0.629 | 0.234 | 0.636 |
| Wilcoxon test for He>Heq | 0.847 | 0.820 | 0.049 | 0.846 | 0.578 | 0.858 | 0.986 | 0.600 |
| | | | | Mechelinki | | | | |
| *StPf1022* | 0.882 | 0.873 | 0.165 | 0.885 | 0.356 | 0.889 | 0.170 | 0.596 |
| *PL142* | 0.914 | 0.884 | 0.068 | 0.908 | 0.402 | 0.918 | 0.230 | 0.755 |
| *FLAG5-83* | 0.957 | 0.948 | 0.264 | 0.960 | 0.309 | 0.964 | 0.104 | 0.442 |

| | | | | | | | | |
|---|---|---|---|---|---|---|---|---|
| *FLAG4-71* | 0.939 | 0.909 | 0.066 | 0.933 | 0.403 | 0.943 | 0.236 | 0.750 |
| *FLAG2-76* | 0.917 | 0.873 | 0.070 | 0.908 | 0.401 | 0.925 | 0.223 | 0.760 |
| *FLAG4-65* | 0.953 | 0.909 | 0.000 | 0.931 | 0.015 | 0.944 | 0.182 | 0.585 |
| *FLAG8-37* | 0.894 | 0.855 | 0.190 | 0.894 | 0.422 | 0.915 | 0.091 | 0.340 |
| *Nplaf_33* | 0.861 | 0.831 | 0.367 | 0.874 | 0.256 | 0.901 | 0.028 | 0.246 |
| *StPf1006* | 0.953 | 0.933 | 0.082 | 0.949 | 0.441 | 0.955 | 0.301 | 0.853 |
| *StPf1001* | 0.801 | 0.890 | 0.026 | 0.919 | 0.001 | 0.934 | 0.001 | 0.375 |
| *FLAC4-67* | 0.949 | 0.930 | 0.105 | 0.945 | 0.459 | 0.954 | 0.221 | 0.757 |
| *FLAC4–69* | 0.890 | 0.857 | 0.257 | 0.893 | 0.370 | 0.916 | 0.060 | 0.515 |
| *PL167* | 0.868 | 0.824 | 0.031 | 0.859 | 0.002 | 0.885 | 0.167 | 0.599 |
| *StPf1004* | 0.716 | 0.672 | 0.165 | 0.694 | 0.356 | 0.711 | 0.170 | 0.596 |
| *StPf1003* | 0.685 | 0.646 | 0.068 | 0.685 | 0.402 | 0.698 | 0.230 | 0.755 |
| *StPf1016* | 0.875 | 0.845 | 0.035 | 0.888 | 0.208 | 0.909 | 0.203 | 0.673 |
| *StPf1002* | 0.687 | 0.667 | 0.095 | 0.683 | 0.219 | 0.710 | 0.137 | 0.463 |
| *StPf1015* | 0.436 | 0.525 | 0.279 | 0.554 | 0.339 | 0.560 | 0.060 | 0.293 |
| *StPf1005* | 0.598 | 0.579 | 0.225 | 0.594 | 0.349 | 0.609 | 0.165 | 0.550 |
| Wilcoxon test for He>Heq | 0.767 | 0.813 | 0.039 | 0.840 | 0.638 | 0.855 | 0.996 | 0.574 |
| Słupsk Bank | | | | | | | | |
| *StPf1022* | 0.888 | 0.884 | 0.283 | 0.894 | 0.310 | 0.899 | 0.113 | 0.633 |
| *PL142* | 0.908 | 0.880 | 0.039 | 0.904 | 0.344 | 0.915 | 0.336 | 0.702 |
| *FLAG5-83* | 0.955 | 0.950 | 0.462 | 0.960 | 0.150 | 0.965 | 0.047 | 0.538 |
| *FLAG4-71* | 0.936 | 0.908 | 0.103 | 0.932 | 0.469 | 0.943 | 0.179 | 0.727 |
| *FLAG2-76* | 0.947 | 0.914 | 0.015 | 0.935 | 0.219 | 0.946 | 0.493 | 0.676 |

| | | | | | | | | |
|---|---|---|---|---|---|---|---|---|
| *FLAG4-65* | 0.956 | 0.921 | 0.002 | 0.939 | 0.040 | 0.949 | 0.246 | 0.650 |
| *FLAG8-37* | 0.913 | 0.882 | 0.190 | 0.913 | 0.431 | 0.930 | 0.072 | 0.408 |
| *Nplaf_33* | 0.879 | 0.816 | 0.112 | 0.862 | 0.386 | 0.894 | 0.205 | 0.933 |
| *StPf1006* | 0.955 | 0.932 | 0.039 | 0.949 | 0.370 | 0.955 | 0.383 | 0.879 |
| *StPf1001* | 0.784 | 0.842 | 0.129 | 0.884 | 0.007 | 0.909 | 0.001 | 0.286 |
| *FLAC4-67* | 0.940 | 0.928 | 0.319 | 0.947 | 0.216 | 0.953 | 0.053 | 0.757 |
| *FLAC4–69* | 0.933 | 0.892 | 0.040 | 0.920 | 0.241 | 0.933 | 0.417 | 0.377 |
| *PL167* | 0.832 | 0.799 | 0.049 | 0.820 | 0.247 | 0.831 | 0.192 | 0.620 |
| *StPf1004* | 0.664 | 0.630 | 0.283 | 0.648 | 0.310 | 0.658 | 0.113 | 0.633 |
| *StPf1003* | 0.645 | 0.614 | 0.049 | 0.645 | 0.996 | 0.662 | 0.336 | 0.702 |
| *StPf1016* | 0.890 | 0.835 | 0.009 | 0.881 | 0.130 | 0.913 | 0.370 | 0.663 |
| *StPf1002* | 0.688 | 0.665 | 0.036 | 0.682 | 0.736 | 0.688 | 0.159 | 0.529 |
| *StPf1015* | 0.464 | 0.523 | 0.003 | 0.565 | 0.041 | 0.590 | 0.139 | 0.671 |
| *StPf1005* | 0.600 | 0.596 | 0.076 | 0.607 | 0.378 | 0.613 | 0.294 | 0.506 |
| Wilcoxon test for He>Heq | 0.844 | 0.812 | 0.001 | 0.836 | 0.406 | 0.850 | 0.968 | 0.647 |
| Vistula lagoon | | | | | | | | |
| *StPf1022* | 0.896 | 0.864 | 0.123 | 0.876 | 0.398 | 0.881 | 0.221 | 0.599 |
| *PL142* | 0.935 | 0.888 | 0.110 | 0.912 | 0.426 | 0.922 | 0.184 | 0.698 |
| *FLAG5-83* | 0.960 | 0.947 | 0.162 | 0.959 | 0.365 | 0.964 | 0.228 | 0.460 |
| *FLAG4-71* | 0.938 | 0.910 | 0.085 | 0.933 | 0.432 | 0.943 | 0.215 | 0.739 |
| *FLAG2-76* | 0.915 | 0.879 | 0.136 | 0.911 | 0.420 | 0.927 | 0.154 | 0.658 |
| *FLAG4-65* | 0.952 | 0.919 | 0.035 | 0.949 | 0.248 | 0.949 | 0.210 | 0.502 |
| *FLAG8-37* | 0.894 | 0.865 | 0.286 | 0.901 | 0.322 | 0.920 | 0.059 | 0.340 |

| | | | | | | | | |
|---|---|---|---|---|---|---|---|---|
| *Nplaf_33* | 0.845 | 0.823 | 0.424 | 0.870 | 0.188 | 0.897 | 0.020 | 0.590 |
| *StPf1006* | 0.957 | 0.935 | 0.043 | 0.950 | 0.291 | 0.956 | 0.379 | 0.896 |
| *StPf1001* | 0.784 | 0.887 | 0.020 | 0.917 | 0.001 | 0.932 | 0.002 | 0.388 |
| *FLAC4-67* | 0.945 | 0.933 | 0.320 | 0.948 | 0.276 | 0.956 | 0.123 | 0.736 |
| *FLAC4–69* | 0.888 | 0.871 | 0.338 | 0.904 | 0.232 | 0.923 | 0.033 | 0.460 |
| *PL167* | 0.829 | 0.818 | 0.069 | 0.850 | 0.219 | 0.871 | 0.206 | 0.579 |
| *StPf1004* | 0.691 | 0.682 | 0.123 | 0.702 | 0.398 | 0.714 | 0.221 | 0.599 |
| *StPf1003* | 0.770 | 0.613 | 0.110 | 0.650 | 0.426 | 0.664 | 0.184 | 0.698 |
| *StPf1016* | 0.785 | 0.843 | 0.095 | 0.889 | 0.334 | 0.914 | 0.182 | 0.580 |
| *StPf1002* | 0.690 | 0.669 | 0.043 | 0.685 | 0.285 | 0.701 | 0.134 | 0.421 |
| *StPf1015* | 0.480 | 0.582 | 0.355 | 0.612 | 0.255 | 0.623 | 0.040 | 0.465 |
| *StPf1005* | 0.653 | 0.594 | 0.233 | 0.609 | 0.239 | 0.619 | 0.199 | 0.743 |
| Wilcoxon test for He>Heq | 0.653 | 0.817 | 0.044 | 0.843 | 0.658 | 0.856 | 0.991 | 0.587 |
